# Endolithic Fungal Diversity in Antarctic Oligocene Rock Samples Explored Using DNA Metabarcoding

**DOI:** 10.3390/biology13060414

**Published:** 2024-06-05

**Authors:** Natana G. Rabelo, Vívian N. Gonçalves, Marcelo A. Carvalho, Sandro M. Scheffler, Gustavo Santiago, Paula A. Sucerquia, Fabio S. Oliveira, Larissa P. Campos, Fabyano A. C. Lopes, Karita C. R. Santos, Micheline C. Silva, Peter Convey, Paulo E. A. S. Câmara, Luiz H. Rosa

**Affiliations:** 1 Departamento de Microbiologia, Universidade Federal de Minas Gerais, Belo Horizonte 31270-901, Brazil; natanagr@gmail.com (N.G.R.); viviannicolau@yahoo.com.br (V.N.G.); 2 Departamento de Geologia e Paleontologia, Museu Nacional, Universidade Federal do Rio de Janeiro, Rio de Janeiro 20940-040, Brazil; mcarvalho@mn.ufrj.br (M.A.C.); schefflersm1@gmail.com (S.M.S.); gustavo.santiago@ymail.com (G.S.); 3 Departmento de Geologia, Universidade Federal de Pernambuco, Recife 50740-540, Brazil; psucerquia@gmail.com; 4 Departamento de Geografia, Universidade Federal de Minas Gerais, Belo Horizonte 31270-901, Brazil; fabiosolos@gmail.com (F.S.O.); larissaparaguassu@gmail.com (L.P.C.); 5 Laboratório de Microbiologia, Universidade Federal do Tocantins, Porto Nacional 77500-000, Brazil; fabyanoalvares@gmail.com (F.A.C.L.); karita.cristine@uft.edu.br (K.C.R.S.); 6 Departamento de Botânica, Universidade de Brasília, Brasília 70297-400, Brazil; silvamicheline@gmail.com (M.C.S.);; 7 British Antarctic Survey, NERC, High Cross, Madingley Road, Cambridge CB3 0ET, UK; pcon@bas.ac.uk; 8 Department of Zoology, University of Johannesburg, Auckland Park 2006, South Africa; 9 Millennium Institute Biodiversity of Antarctic and Subantarctic Ecosystems (BASE), Las Palmeras 3425, Santiago 8320000, Chile; 10 Cape Horn International Center (CHIC), Puerto Williams 6350000, Chile; 11 Programa de Pós-Graduação em Fungos, Algas e Plantas, Universidade Federal de Santa Catarina, Florianópolis 88040-900, Brazil

**Keywords:** Antarctica, eDNA, fungi, environmental drivers

## Abstract

**Simple Summary:**

We investigated the environmental DNA fungal diversity present in cores of Oligocene rocks from Antarctica using a DNA metabarcoding approach. After eDNA extraction and analysis, we identified 198 amplicon sequence variants (ASVs) representing the phyla *Ascomycota*, *Basidiomycota*, *Mortierellomycota*, *Chytridiomycota*, *Mucoromycota*, *Rozellomycota*, *Blastocladiomycota*, *Monoblepharomycota*, *Zoopagomycota*, *Aphelidiomycota* (Fungi), and the fungal-like *Oomycota* (Stramenopila). The dominant taxa detected were *Pseudogymnoascus pannorum*, *Penicillium* sp., *Aspergillus* sp., *Cladosporium* sp., *Aspergillaceae* sp., and *Diaporthaceae* sp. We also detected 22 fungal ASVs as intermediate and 170 as minor components of the assigned fungal diversity. The samples displayed a high fungal diversity; however, rarefaction analysis suggested that further diversity remains to be detected. The endolithic fungal community detected contains a rich and complex mycobiome comprising taxa with different lifestyles, comparable to the diversity reported in other Antarctic habitats. Our results suggest the need for further research to develop strategies for isolating these fungi into culture for evolutionary, physiological, and biogeochemical studies, and to assess their potential roles in biotechnological applications.

**Abstract:**

In this study, we evaluated the fungal diversity present associated with cores of Oligocene rocks using a DNA metabarcoding approach. We detected 940,969 DNA reads grouped into 198 amplicon sequence variants (ASVs) representing the phyla *Ascomycota*, *Basidiomycota*, *Mortierellomycota*, *Chytridiomycota*, *Mucoromycota*, *Rozellomycota*, *Blastocladiomycota*, *Monoblepharomycota*, *Zoopagomycota*, *Aphelidiomycota* (Fungi) and the fungal-like *Oomycota* (Stramenopila), in rank abundance order. *Pseudogymnoascus pannorum*, *Penicillium* sp., *Aspergillus* sp., *Cladosporium* sp., *Aspergillaceae* sp. and *Diaporthaceae* sp. were assessed to be dominant taxa, with 22 fungal ASVs displaying intermediate abundance and 170 being minor components of the assigned fungal diversity. The data obtained displayed high diversity indices, while rarefaction indicated that the majority of the diversity was detected. However, the diversity indices varied between the cores analysed. The endolithic fungal community detected using a metabarcoding approach in the Oligocene rock samples examined contains a rich and complex mycobiome comprising taxa with different lifestyles, comparable with the diversity reported in recent studies of a range of Antarctic habitats. Due to the high fungal diversity detected, our results suggest the necessity of further research to develop strategies to isolate these fungi in culture for evolutionary, physiological, and biogeochemical studies, and to assess their potential role in biotechnological applications.

## 1. Introduction

Antarctic ecosystems experience polyextremophilic environmental stresses and offer unique opportunities to discover and study extremophilic organisms [1]. While more than 99.6% of the Antarctic continent is currently permanently covered by ice and snow, the remaining snow-/ice-free area provides diverse habitats including soils and sediments, fresh waters, vegetation, and rocks, hosting a diversity of organisms [2]. These are characterized by the presence of complex microbial communities, including fungi and their allies, that are present in virtually all Antarctic ecosystems, where they display multiple ecological roles [3,4,5].

Fungal diversity is high in many ecosystems, where they play important ecological roles. Meanwhile, these ecosystems are also facing the impacts of contemporary climatic and other environmental changes [5,6,7,8]. Antarctic fungal communities include taxa highly adapted to survival in habitats that experience rapid temperature fluctuations, along with prolonged desiccation, high levels of solar radiation and lack of nutrients [5]. Rocks can provide a range of habitats that are occupied by different fungal groups, which can live directly on the exposed surfaces of rocks, as well as forming hypolithic communities on the more protected undersurfaces of translucent rocks (e.g., quartz, gypsum) [3]. Fungi living within rocks are known as endoliths, taking advantage of small and even microscopic fissures within the rocks (chasmoendoliths) or, at the most extreme, the spaces between the individual crystals which comprise the rock (cryptoendoliths) [3,7,9,10,11,12,13,14,15].

Despite their importance, few studies of endolithic fungal diversity in Antarctica have been reported until recently, most using traditional culturing methods [16]. Only the studies published by [15,17] used a high-throughput sequencing (HTS) approach to analyse the unculturable portion of the Antarctic endolithic fungal community. However, there are differences in the fungal diversity profiles between culturable and non-culturable fungi. The use of traditional culturing methods revealed the presence of a limited diversity of taxa dominated by the phyla *Ascomycota* and *Basidiomycota* [18]; in contrast, metabarcoding studies have shown high taxa richness again dominated by *Ascomycota* and *Basidiomycota*, but also with the presence of the phyla *Mortierellomycota* and *Mucoromycota* [15,17].

The South Shetland Islands archipelago is composed of Mesozoic and Cenozoic volcanic and volcaniclastic rocks. These rocks were formed during the Paleogene and Neogene periods (~23 to 66 Mya). The Oligocene age, in particular, is represented by numerous lithostratigraphic units (formations) [18], including the study site utilized here, the Polonez Cove Formation (33–25.7 Mya) [19]. The Oligocene (~34–23 Mya) was a period marked by significant climatic and evolutionary changes in the Antarctic region, with the continent undergoing cooling, eventually resulting in the formation of extensive continental ice sheets which reached their maximum extent in the Miocene and remain to the present day [20,21,22,23,24,25]. These considerable environmental changes were accompanied by a drastic reduction in Antarctic terrestrial diversity [21,22]. The sediments of the Polonez Cove Formation provide evidence of a reduced vegetation characterized by a sparse tundra flora vegetation, with limited herbaceous plants and rare podocarp conifers and southern beech (*Nothofagus*) [24].

In recent years, Antarctic fungal diversity studies have taken advantage of the development of environmental DNA (eDNA) metabarcoding approaches, which have revealed the potential presence of previously unrecognized and complex communities present in different substrates and habitats, as indicated by high levels of assigned sequence diversity [26,27,28,29]. Although the importance of endolithic habitats in the search for microbial diversity at the edge of life has been recognized, few studies on the fungal diversity of Antarctic endolithic ecosystems have been reported until recently, as noted above. To date, no studies of the endolithic fungal diversity present in ancient Antarctic rocks have been conducted. Here, we used metabarcoding to detect and characterize the endolithic fungal community in Oligocene rocks obtained from King George Island, South Shetland Islands, Antarctica.

## 2. Materials and Methods

### 2.1. Sample Strategy and Processing

**Figure 1:**
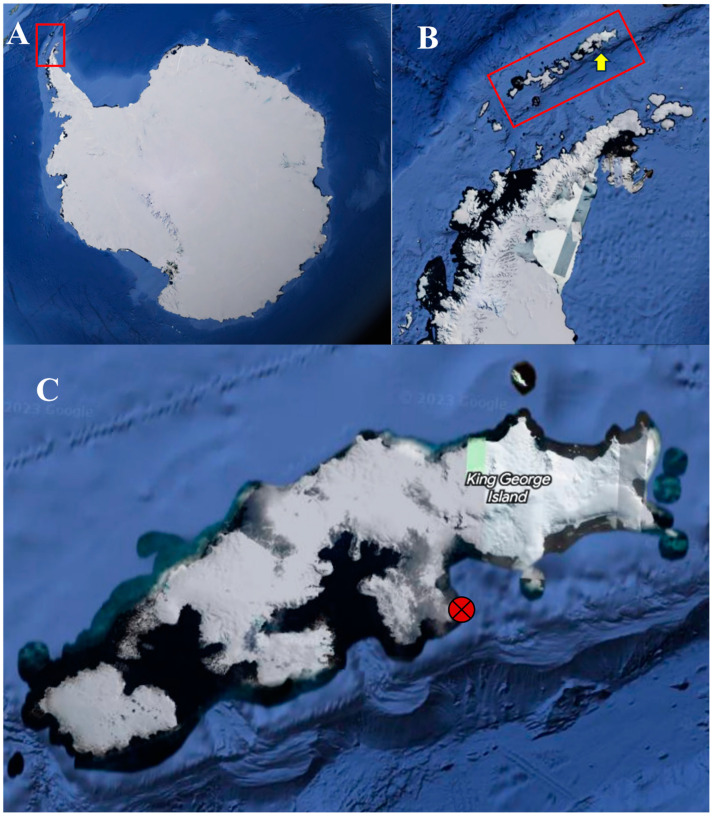
Satellite images of the study region from which the rock samples analysed were obtained (https://www.google.com.br/maps/, accessed on 28 February 2024). (**A**) Antarctica, with the Antarctic Peninsula indicated within the red rectangle, (**B**) South Shetland Islands indicated within red rectangle and King George Island indicated by yellow arrow, (**C**) King George Island with the Lions Rump region highlighted by the red circle. Images obtained from Google Earth Pro, 2019 (https://earth.google.com, accessed on 28 February 2024).

**Figure 2:**
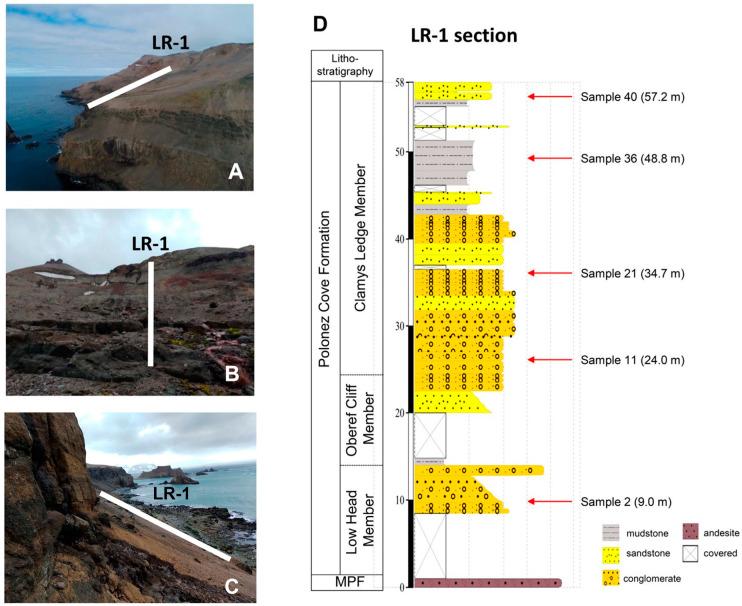
Lithostratigraphic scheme of the Mazurek Point Formation at Lions Rump, King George Island. (**A**–**C**): sections where the samples were obtained. Photographs of the limits (base and top), where the samples were collected. (**D**): Geological interpretation of the Formation; MPF = Mazurek Point Formation. Images by Gustavo Santiago. Samples 9.0 m (sample 2), 24.0 m (sample 11), 34.7 m (sample 21), 48.8 m (sample 36) and 57.2 m (sample 40).

The rock material examined in this study derives from a section coded as LR-1 = Lions Rump-1 located in the Mazurek Point Formation [30] at Lions Rump region (62°08′30.81″ S 58°07′34.25″ W), King George Island, South Shetland Islands, during the austral summer of 2021/22 (Figure 1), which, according to paleobotanic studies, represents rock from the Oligocene epoch. Section LR-1 has a thickness of ~58 m and five rock samples were obtained at 9 m (sample-S2), 24 m (S11), 34.7 m (S21), 48.8 m (S36) and 57.2 m (S40) using a geological hammer. The rock samples (Figure 2) were stored in sterile Whirl Pack bags (Nasco, Ft. Atkinson, NH, USA) and frozen at −20 °C until processing for eDNA at the Federal University of Minas Gerais, Brazil. There, the samples were first fragmented into approximately 1 cm^3^ using a miniature drill device (Storm Feel-0001, Gumroad, Sumaré, Brazil) with diamond drills, one for each sample, and autoclaved before extraction [25]. After that, these fragments were pulverized with the aid of a previously sterilized agate mortar. The entire process was carried out in a laminar flow cabinet to avoid external contamination.

### 2.2. Stratigraphy

The Polonez Cove Formation is a continuous 2 km-long cliff section between Low Head and Lions Rump. The formation consists of a basal unit dominated by glacigenic diamictite, overlain by a series of basalt-sourced sedimentary and dacitic volcanic units [31]. The Polonez Cove Formation has been divided into six members [23]: Krakowiak Glacier; Low Head; Bayview; Siklawa; Oberek Cliff; and Chlamys Ledge. These members have been interpreted as: (1) sparsely fossiliferous, planar-bedded, generally fine-grained sediments (mainly siltstones and fine sandstones) formed in relatively deep water; (2) locally fossiliferous coarse epiclastic fan deltas sourced in recently active volcanism (subunits of the Low Head and Oberek Cliff members); and (3) gravelly conglomeratic sediments with prominent channels and cross-stratification, typically highly fossiliferous and characteristic of deposition under relatively shallow-water high-energy conditions (other subunits of the Low Head and Oberek Cliff members) [24].

### 2.3. Geochemical Analysis

Analysis of chemical element concentrations in the LR-1 bulk sediment rock samples (*n =* 1) was performed using X-ray fluorescence (XRF) at the National Museum (Rio de Janeiro, Brazil), utilizing a Bruker Tracer 5i Handheld XRF instrument (Bruker, Billerica, MA, USA). The data obtained were interpreted in the context of elemental ratios, specifically the Fe/Ca ratio, to deduce the presence of terrigenous inputs. This is because iron is more prevalent in terrestrial materials such as soils and rocks, whereas calcium tends to be the dominant element in marine environments, owing to the abundance of calcium-rich minerals such as calcite and aragonite. The Sr/Ca ratio was used to estimate relative sample temperatures, where higher values indicate warmer waters (higher Sr concentration) and lower values suggest colder waters (higher Ca concentration).

### 2.4. Petrographic Analyses

Thin sections were made with the undisturbed samples, and optical microscopic investigations were carried out using a Zeiss trinocular optical microscope (Axiophot model) with an integrated digital camera, to identify the types of rocks and their features, in particular any crack/fissure patterns. Rock porosity is an important part of the formation of habitats suitable for microbial colonization [13,31]. We follow [9] for void system description. Samples 11, 36, and 40 (*n =* 1), which displayed the best structural conditions for petrographic analysis, were selected to analyse the relationship between the degree of rock porosity and its chemical alteration, in order to identify whether the rocks showed any evidence of recent colonization by fungi, as some fungal species (particularly those that can become lichenized) are known to exude chemicals that can alter the rock minerals present [32]. For this, we used a scanning electron microscope (SEM, QUANTA FEI 3D) coupled with an energy dispersive system (EDS), operated with an acceleration potential of 15 kV and a current of 20 nA. The following elements were determined in the analysis: Na, K, Mg, Ca, Fe, Al and Si. The thin sections were metalized with carbon films, and this element was used as an indicator of porosity in the microchemical maps produced.

### 2.5. DNA Extraction, Illumina Library Construction and Sequencing

One sub-sample (approximately 100 g) of each of sample S2, S11, S21, S36 and S40 (five for each sample) was pulverized as described by [31]. Total DNA was extracted from these using the FastDNA Spin Kit for Soil (MPBIO, Solon, OH, USA) under strict contamination control conditions, following the manufacturer’s instructions. DNA quality was analysed using agarose gel electrophoresis (1% agarose in 1 × Trisborate-EDTA) and then quantified using the Quanti- iT™ Pico Green dsDNA Assay (Invitrogen, Carlsbad, CA, USA). Extracted DNA was used as template for generating PCR amplicons.

The internal transcribed spacer 2 (ITS2) of the nuclear ribosomal DNA was used as a DNA barcode for molecular species identification [27,33,34,35]. PCR amplicons generated using the universal primers ITS3 and ITS4 [36] were sequenced commercially by Macrogen Inc. (Seoul, Republic of Korea) using high-throughput paired-end sequencing (2 × 300 bp) on a MiSeq System (Illumina, San Diego, CA, USA), using the MiSeq Reagent Kit v3 (600 cycles). Library construction and DNA amplification were performed using the Library kit Herculase II Fusion DNA Polymerase Nextera XT Index Kit V2 (Illumina, San Diego, CA, USA), following Illumina 16S Metagenomic Sequencing Library Preparation Part #15044223 Rev. B protocol.

### 2.6. Data Analysis and Fungal Identification

Quality analysis was carried out using BBDuk v. 38.87 in BBmap software [37] with the following parameters: Illumina adapters removing (Illumina artefacts and the PhiX Control v3 Library); ktrim = l; k = 23; mink = 11; hdist = 1; minlen = 50; tpe; tbo; qtrim = rl; trimq = 20; ftm = 5; maq = 20. The remaining sequences were imported to QIIME2 version 2021.4 (https://qiime2.org/, accessed on 28 February 2024) for bioinformatics analyses [38]. The qiime2-dada2 plugin was used for filtering, dereplication, turn paired-end fastq files into merged, and to remove chimeras, using default parameters [39]. Taxonomic assignments were determined for amplicon sequence variants (ASVs) in three steps. First, ASVs were classified using the qiime2-feature-classifier [40] classify-sklearn against the UNITE Eukaryotes ITS database version 8.3 [41]. Second, remaining unclassified ASVs were filtered and aligned against the filtered NCBI non-redundant nucleotide sequences (nt) database (October 2021) using BLASTn [42] with default parameters; the nt database was filtered using the following keywords: “ITS1”, “ITS2”, “Internal transcribed spacer”, and “internal transcribed spacer”. Third, output files from BLASTn [42] were imported to MEGAN6 [43] and taxonomic assignments were performed using the “megan-nucl-Jan2021.db” mapping file with default parameters and trained with Naive Bayes classifier and a confidence threshold of 98.5%. Taxonomic profiles were plotted using the Krona [44]. The heatmap of ASV abundance and clustering analysis were performed using Heatmapper [45]; clustering analysis was performed using the following parameters: average linkage, Spearman’s rank correlation, and Z-score among samples for each ASV. Sequences have been submitted to GenBank under the accession numbers SAMN37305760-SAMN37305772.

Many factors, including extraction, PCR and primer bias, can affect the number of reads obtained [46], and thus lead to misinterpretation of absolute abundances [47]. However, ref. [48] concluded that such biases did not affect the proportionality between reads and cell abundance, implying that more reads are linked with higher abundance [49,50]. Therefore, for comparative purposes, we used the number of reads as a proxy for relative abundance. Fungal classification followed [51,52], MycoBank (http://www.mycobank.org, accessed on 28 February 2024) and the Index Fungorum (http://www.indexfungorum.org, accessed on 28 February 2024).

### 2.7. Fungal Diversity

The relative abundances of the ASVs were used to quantify the fungal taxa present in the samples; fungal ASVs with relative abundance > 10% were considered dominant, those between 1% and 10% as intermediate, and those with <1% as minor (rare) components of the fungal community [27]. The relative abundances were used to quantify taxon diversity, richness, and dominance, using the following indices: (i) Fisher’s α, (ii) Margalef’s and (iii) Simpson’s, respectively. In addition, species accumulation rarefaction curves were obtained using the Mao Tao index. All results were obtained with 95% confidence, and bootstrap values were calculated from 1000 replicates using the PAST 1.90 [53]. Venn diagrams were prepared following [54] to visualise the fungal assemblages present in the different sampling areas.

## 3. Results

### 3.1. Fungal Identification

**Figure 3:**
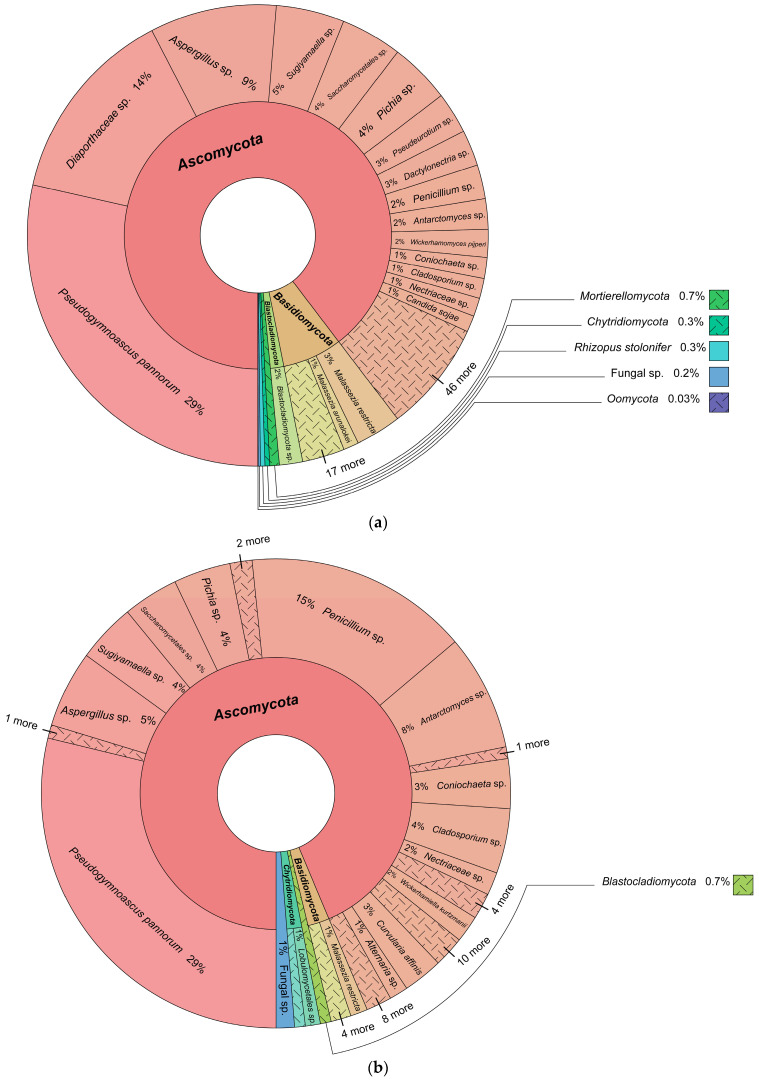

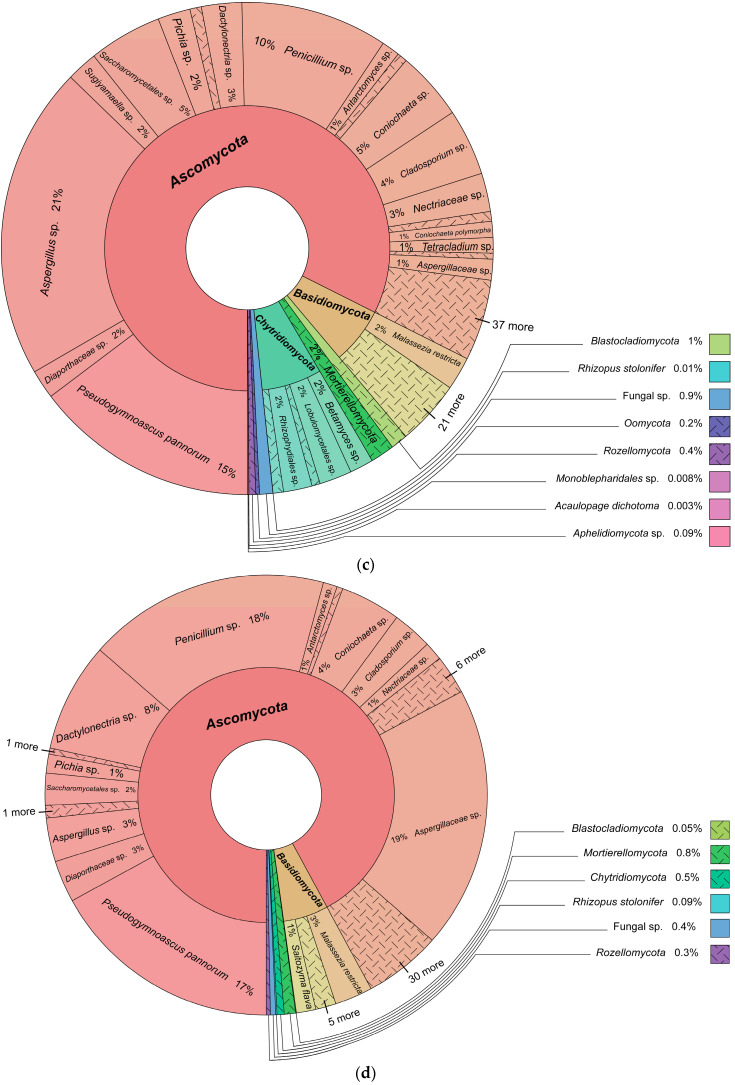

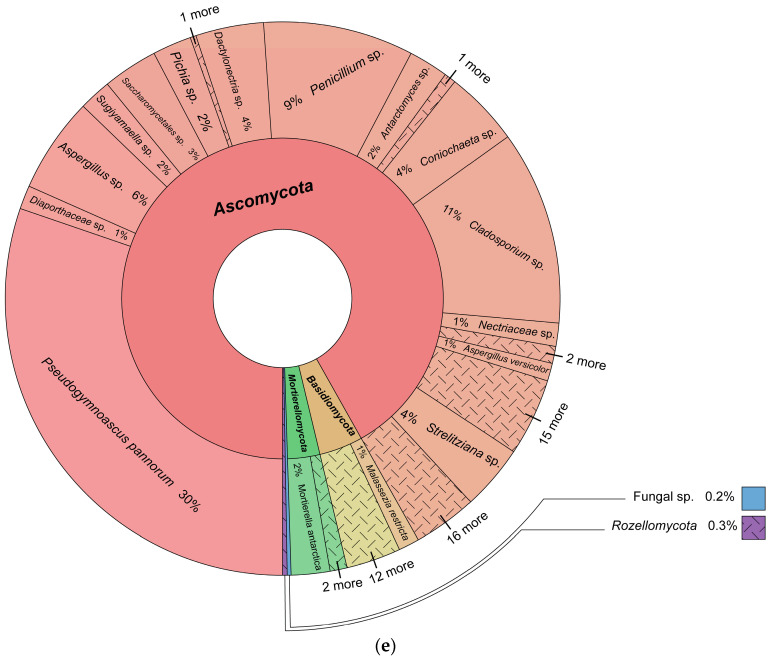
Krona charts of the fungal assemblages detected from the five different rock samples obtained at Lions Rump, King George Island, South Shetland Islands. (**a**) Sample 2, (**b**) sample 11, (**c**) sample 21, (**d**) sample 36, and (**e**) sample 40, each referring to a specific fungal community. ‘More’ indicates the presence of other different taxa.

A total of 940,969 fungal DNA reads were obtained, which were assigned to 198 amplicon sequence variants (ASVs) (Figure 3, Supplementary Table S1). *Ascomycota* was the dominant phylum, followed by *Basidiomycota*, *Mortierellomycota*, *Chytridiomycota*, *Mucoromycota*, *Rozellomycota*, *Blastocladiomycota*, *Monoblepharomycota*, *Zoopagomycota*, *Aphelidiomycota* (Fungi) and the fungal-like *Oomycota* (Stramenopila), in rank order (Supplementary Figure S1). *Pseudogymnoascus pannorum*, *Penicillium* sp., *Aspergillus* sp., *Cladosporium* sp., *Aspergillaceae* sp. and *Diaporthaceae* sp. were the most abundant taxa (relative abundance ≥ 10%). In addition, 22 fungal ASVs displayed intermediate abundance, and 170 formed minor components of the assigned fungal community.

### 3.2. Fungal Diversity and Distribution

**Figure 4:**
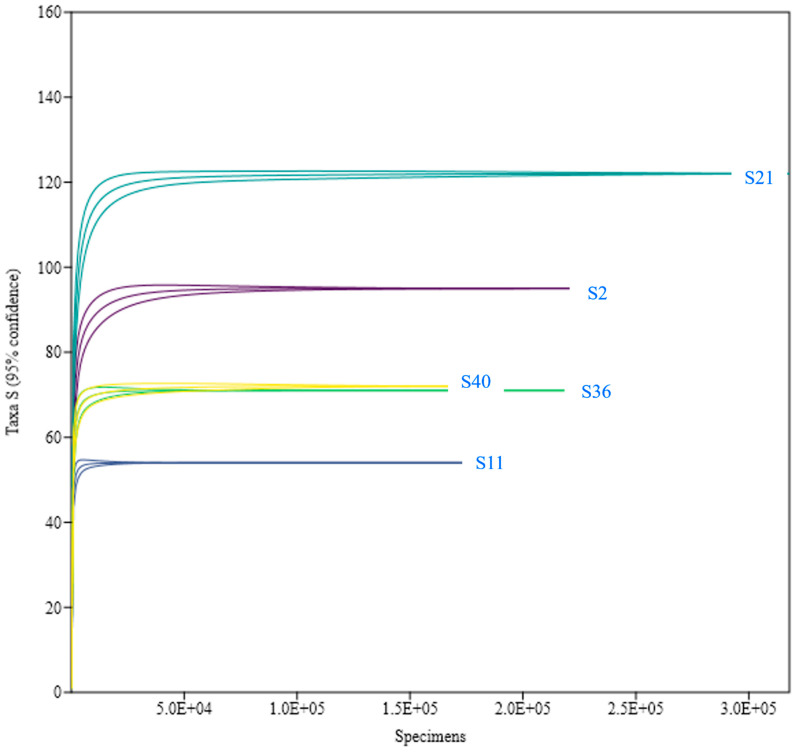
Rarefaction curves (Mao Tao index) for fungal assemblages detected from each of the five rock samples. S2 (sample 2), S11 (sample 11), S21 (sample 21), S36 (sample 36) and S40 (sample 40). Results obtained using the PAST computer program 1.90.

**Figure 5:**
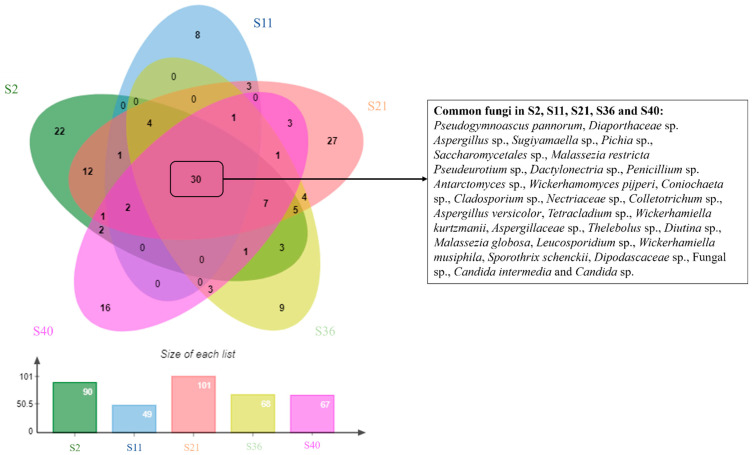
Venn diagram illustrating fungal diversity across the five samples.

The Mao Tao rarefaction curves reached asymptote for all fungal assemblages from the different sites, indicating that the majority of the diversity was detected (Figure 4). The diversity indices varied among the fungal assemblages for the five sites sampled (Table 1). The highest diversity assemblages were detected in S21 and S2, whilst those from S11, S36 and S40 displayed the lowest values. Among the 198 fungal ASVs assigned, 30 (15%) were detected in all five samples (Figure 5).

### 3.3. Geochemical Results

**Figure 6:**
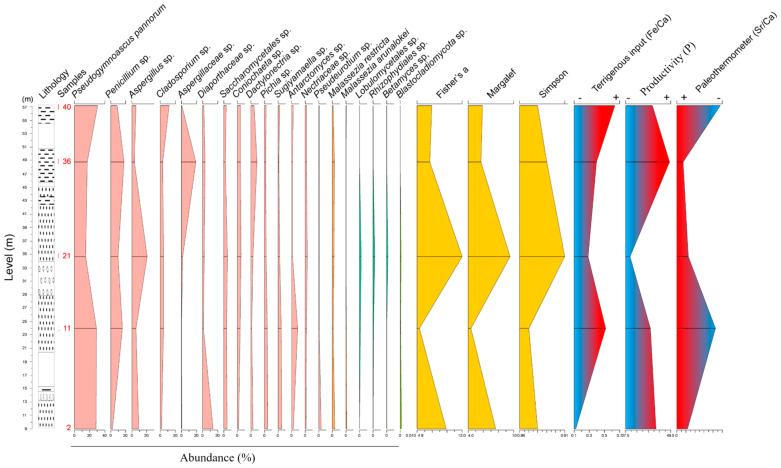
Comparison across the lithology of the most abundant fungal taxa, diversity indices, terrigenous input (Fe/Ca), productivity (P) and paleothermometer (Sr/Ca) data detected in samples 2, 11, 21, 36 and 40.

X-ray fluorescence results (Table 1) revealed differences in the chemical compositions of the five samples. The major chemical elements were Si (with an average of 42.8%), Fe (37.2%) and Al (7.3%). Other elements were also present in minor proportions in the samples, including Mg, P, and Ti (Table 1). The curves of the Fe/Ca and Sr/Ca ratios exhibited similar patterns, with the lowest concentrations found in sample S2 and the highest in sample S40. Meanwhile, the phosphorus (P) concentration, indicative of productivity, exhibited its lowest levels in sample S21 and highest in S36 (Figure 6).

The stratigraphic distribution of the relevant fungal ASVs revealed a correlation between high diversity (S21) in conjunction with rocks formed during a period of reduced terrigenous input and higher temperatures. Conversely, lower diversity values (S11) were associated with rocks formed during periods of moderate to high terrigenous input and lower temperatures. (Figure 6). Specifically, the abundance curve of *P. pannorum* followed the same pattern as the curve of terrigenous input (Figure 6), suggesting a connection between this species and rocks whose formation and properties are related to terrigenous input. Conversely, the species *Aspergillus* sp. and *Penicillium* sp. were associated with rocks formed during periods of higher temperatures.

### 3.4. Petrographic Analyses

**Figure 7:**
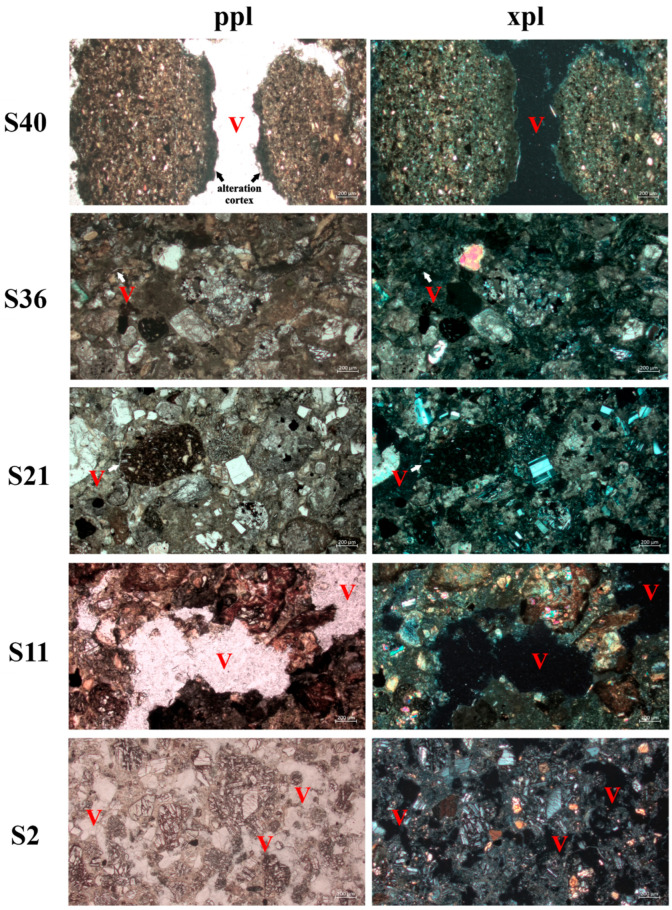
Petrographic microscope photomicrographs in plane polarized light (**ppl**) and crossed polarized light (**xpl**) of the samples in section LR-1. **V** = voids.

The photomicrographs show that the samples had different degrees and patterns of micro-fissure formation (Figure 7). Samples S2, S11, and S40 were the most fragmented, with voids that varied from flat and curved planes to a simple packing void system. In conglomerates (S2 and S11), fissure formation had occurred through the fragmentation of the matrix and the release of rounded clasts. The sandstone rock (S40) was also fractured, with some rotated clasts. Samples S21 and S36 showed the lowest degree of fragmentation, with some areas with poorly connected flat voids. The less fragmented samples were those that presented the highest fungal diversity values (Figure 7).

Our analyses identified little evidence of chemical weathering, with the presence of an alteration cortex observed under optical microscopy only in sample S2 (sandstone). In the conglomerate rocks, backscattered electron microscopy images and microchemical maps (Supplementary Figures S2–S4) indicated that zones of geochemical alteration were not evident in the void systems present. Comparing samples S11, S36, and S40, there were differences in void size, but no evidence of chemical alteration. The specific location of carbon indicates the presence of pores, as the thin sections were metallized with graphite, with the numbers increasing from samples S36 and S40 to S11, respectively 20.33%, 25.66%, and 44.05% carbon (Supplementary Figures S2d, S3d, and S4d).

### 3.5. Assignment to Fungal Lifestyles

**Figure 8:**
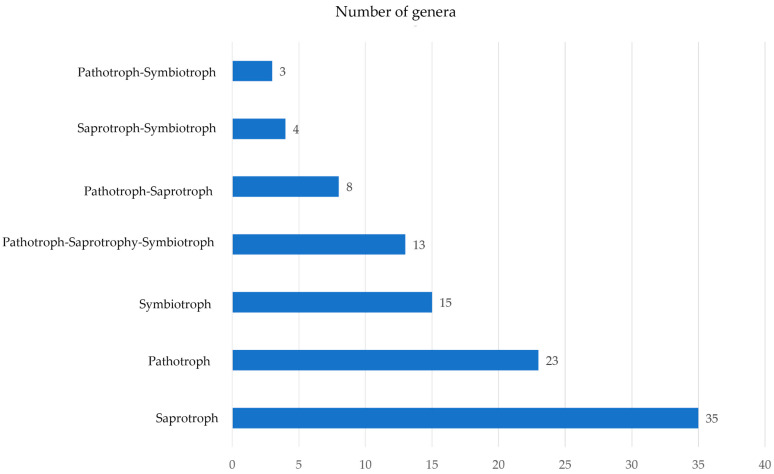
Assignment to fungal lifestyles of the ASVs detected in Oligocene rock samples obtained from the South Shetland Islands, maritime Antarctic.

**Figure 9:**
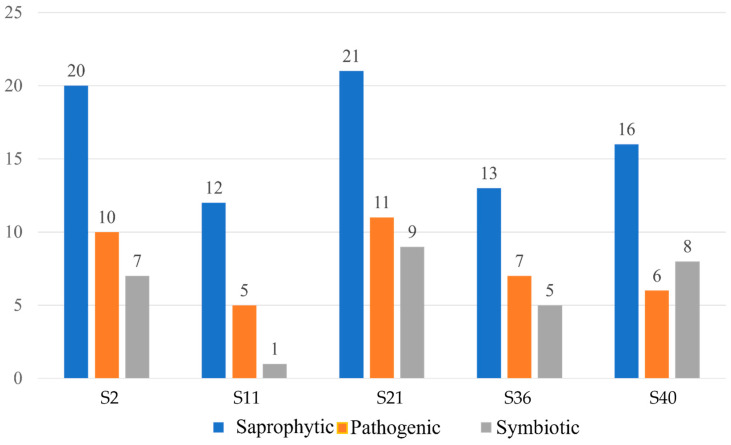
Diversity of saprophytic, pathogenic and symbiotic fungal taxa in the different Oligocene rock samples analysed from the South Shetland Islands, maritime Antarctic.

The 101 fungal ASVs genera detected were assigned to fungal lifestyles using FunGuild (Supplementary Table S2). The majority of assignments were to saprotrophic, pathogenic, and symbiotic taxa, with some displaying multiple ecological roles (Figure 8). The fungal genera included animal and plant pathogens, soil, wood, litter and undefined saprophytic taxa, endophytes, and lichenized fungi. Considering only ASVs classified to a single ecological status (Figure 9), in four of the samples saprophytic taxa dominated, followed by pathogenic and symbiotic taxa, while, in sample S40, symbiotic taxa were more diverse than pathogenic taxa.

## 4. Discussion

### Fungal Taxonomy, Fungal Diversity, and Fungal Lifestyle

Existing studies of endolithic fungal communities present in Antarctic rocks have involved their direct observation in the substrate, cultivation techniques, or culture-independent methods [13]. Rocks from Antarctic can be considered a unique microhabitat for life forms, providing a natural habitat for rock-inhabiting microbes, including fungal taxa [7,13,17]. In the extreme conditions across Antarctica, endolithic fungi present within rocks are dominated by the phyla *Ascomycota* and *Basidiomycota*, as revealed by studies using traditional culturing methods [13].

The use in this study of metabarcoding to detect fungal eDNA associated with Antarctic Oligocene rocks revealed the presence of eDNA assigned to the widespread and common phyla *Ascomycota* and *Basidiomycota*. It also detected members of the less commonly reported *Mortierellomycota*, *Chytridiomycota*, and *Mucoromycota*, as well as cryptic fungi from the phyla *Rozellomycota*, *Blastocladiomycota*, *Monoblepharomycota*, *Zoopagomycota*, *Aphelidiomycota*, and fungal-like *Oomycota* (Stramenopila). Among the ASVs assigned, 74 were resolved only at higher hierarchical taxonomic levels (family, class, order, and phylum), suggesting that they might represent undescribed fungi or taxa not currently included in publicly accessible databases, which deserve further taxonomic studies.

In a study carried out in a polar desert region of continental Antarctica, rocks were analysed using DNA metabarcoding, reporting 262,268 fungal DNA reads grouped into 517 fungal ASVs dominated by the phyla *Ascomycota*, *Basidiomycota*, *Mortierellomycota*, and *Mucoromycota*, in abundance order [17]. These results showed similarity to those obtained in our study [55,56,57], in which *Ascomycota* and *Basidiomycota* represented the dominant phyla, with *Mortierellomycota* and *Mucoromycota* as minor components. However, in the current study, we detected taxa from further phyla including *Chytridiomycota*, *Rozellomycota*, *Blastocladiomycota*, *Monoblepharomycota*, *Zoopagomycota*, *Aphelidiomycota*, and the fungal-like Oomycota (Stramenopila). The dominant genera detected in the current study (*Pseudogymnoascus*, *Penicillium*, *Aspergillus*, and *Cladosporium*) were similar to those reported in [17].

Endolithic microbes, including those present in Antarctica, can exist in a dormant form alongside non-viable cells that retain their morphological integrity [58]. It is therefore important to recognize that the metabarcoding approach can detected only the presence of DNA and does not confirm viability. The petrographic analysis confirms the presence of microchannels within the rocks, and we assume that spores, hyphal fragments, or resistant structures might be recovered from such structures, as proposed by [13]. 

Amongst the taxa assigned, *Pseudogymnoascus pannorum* and representatives of the genera *Penicillium*, *Aspergillus*, and *Cladosporium* were the most abundant. The genus *Pseudogymnoascus* (syn. *Geomyces*) includes many psychrophilic and psychrotolerant species commonly present in Antarctic terrestrial and marine habitats [5]. Although the genus is widely distributed in cold environments globally [59,60], it is particularly well represented in Antarctica, with reports from habitats including soils [59,61,62], in association with plants [63], marine macroalgae [64,65], lichens [66], sponges [67] and in freshwater lakes [1,68]. *Pseudogymnoascus pannorum* was reported to produce high levels of protease at low temperatures [69], which may contribute to its occurrence, dominance, and capability to survive in the extreme conditions of Antarctica.

Representatives of the genera *Penicillium*, *Aspergillus*, and *Cladosporium* are found globally, including some truly cosmopolitan species. They have been reported from multiple Antarctic habitats, including soils [62,70] and permafrost [71,72]. Taxa of *Penicillium*, *Aspergillus*, and *Cladosporium* have been reported as endolithic microbes in rocks across various environments. Within Antarctic rocks, taxa from these genera have been identified as dominant [13,17,73]. Recent metabarcoding studies in Antarctica have reported eDNA assignments to members of *Pseudogymnoascus*, *Penicillium*, *Aspergillus*, and *Cladosporium* from habitats including mosses, soils [26,72,74], snow [75], rock surfaces [55], marine environments [76], and lake sediments [29].

The diversity indices and similarity among the fungal assemblages varied. The highest diversity values were detected in the fungal assemblages of S21 and S2, followed by those of S11, S36, and S40. These results might be explained by differences in the formation of the rock samples. Rock fragmentation in periglacial environments occurs primarily through cryoclastic processes [77,78] and is influenced by the type of rock, its position in the relief, exposure time, and the intensity of freezing and thawing cycles [79,80,81]. In the South Shetland Islands, some studies have shown that sedimentary rocks are more easily fragmented than igneous rocks [82]. This is consistent with the high degree of fragmentation observed in the samples studied here, which included conglomerates and sandstones. The latter are also more affected by cryoturbation, because of their greater compositional homogeneity. As they are progressively fragmented, the rocks can become chemically altered along the fissures, or fragments may be physically removed by water or ice activity. However, chemical modification was not evident in the rock samples analysed here (Supplementary Figures S2–S4). Therefore, the chemical change might be promoted by biological action as proposed by [31,83,84,85].

Although this study is based on a small sample size, samples S2, S11, and S40 were the most fragmented rocks, while samples S21 and S36 displayed the lowest degree of fragmentation. We therefore expected that samples S21 and S36 would present low diversity indices and the most fragmented samples would have the highest diversity indices. However, fragmentation did not show a correlation with eDNA fungal assemblage diversity. Rather, our data suggest a possible link between (undefined) rock properties developed at their time of formation under the influence of different environmental temperatures (as indicated by Sr/Ca ratio) and their subsequent, much more recent, colonization by endolithic fungi. 

The ecological functional group assignments in the current study are similar to our recent metabarcoding studies of fungal communities in various Antarctic habitats [26,28,29,72,74,86,87]. Our ASV assignments suggest the presence of a diverse fungal community associated with the Oligocene rock samples analysed. The dominance of saprophytic fungi is consistent with the studies of [57,88], which suggest that fungi can colonize and establish in cold environments, slowly degrading the limited organic matter available, releasing nutrients, and making them available to other organisms.

## 5. Conclusions

The use of a metabarcoding approach led to detection of a diverse endolithic fungal community associated with the Oligocene rock samples examined. The endolithic fungal community detected contains a rich and complex mycobiome comprising taxa with different lifestyles, comparable with the diversity reported in recent studies of a range of Antarctic habitats. Due to the high fungal diversity detected, our results highlight the need for further research to develop strategies to isolate these fungi in culture for evolutionary, physiological, and biogeochemical studies, and to assess their potential role in biotechnological applications.

## Figures and Tables

**Table 1:** Chemical elements present in the rock samples analysed and diversity indices of fungal assemblages detected associated with the different rock samples.

	Sample				
Chemical Elements *	S2	S11	S21	S36	S40
Mg	4.13	4.89	3.40	<LOD	<LOD
Ca	4.14	18.07	14.92	7.97	2.76
Al	5.87	7.89	8.16	8.83	7.88
Si	44.06	45.62	38.68	49.14	44.61
P	0.14	2.54	1.13	0.45	0.13
S	0.75	2.90	1.64	0.66	0.704
Ti	1.81	0.79	2.15	1.96	2.43
V	0.05	<LOD	0.05	<LOD	0.0858
Cr	0.21	0.15	0.215	0.27	0.11
Mn	0.64	2.23	0.59	1.17	2.15
Fe	40.03	29.72	41.83	34.27	38.25
Co	0.12	<LOD	0.14	<LOD	0.0794
Ni	0.52	0.31	0.22	0.33	0.21
Cu	0.1	0.1	0.11	0.02	0.09
Zn	0.05	0.05	0.06	0.04	0.06
Zr	0.34	0.58	0.45	0.19	0.38
Pd	1.11	2.06	1.16	0.87	1.08
**Altitude above sea level (m)**	9.0	24.0	34.7	48.8	57.2
**Diversity indices**					
Number of amplicon sequence variants (ASVs)	90	49	101	68	67
Number of assigned ASVs	225	177,098	319,448	219,426	172,971
Fisher’s α	9.42	5.17	11.97	6.84	7.13
Margalef	7.63	4.39	9.55	5.69	5.89
Simpson	0.88	0.87	0.91	0.89	0.88

* The concentration measured in percentage. LOD = limit of detection, ASV = amplicon sequence variant.

## Data Availability

The datasets generated and/or analysed during the current study are available in the NCBI repository under the codes SAMN37305760-SAMN37305772, which can be accessed in https://www.ncbi.nlm.nih.gov/. Data are provided in the article.

## Supplementary Materials

Please add this part: The following supporting information can be downloaded at: https://www.mdpi.com/article/10.3390/biology13060414/s1. Refs. [89,90,91,92,93,94,95,96,97,98,99,100,101,102,103,104,105,106,107,108,109,110,111,112,113,114,115,116,117,118,119] are cited in Supplementary Materials.
