# Supplementary material for: Endolithic Fungal Diversity in Antarctic Oligocene Rock Samples Explored Using DNA Metabarcoding
Source: Biology (Basel). 2024 Jun 5;13(6):414. doi: 10.3390/biology13060414 (PMC11200754; doi:10.3390/biology13060414)
Supplement: Supplementary file 1 [file biology-13-00414-s001.zip › biology-2995452-supplementary.pdf]

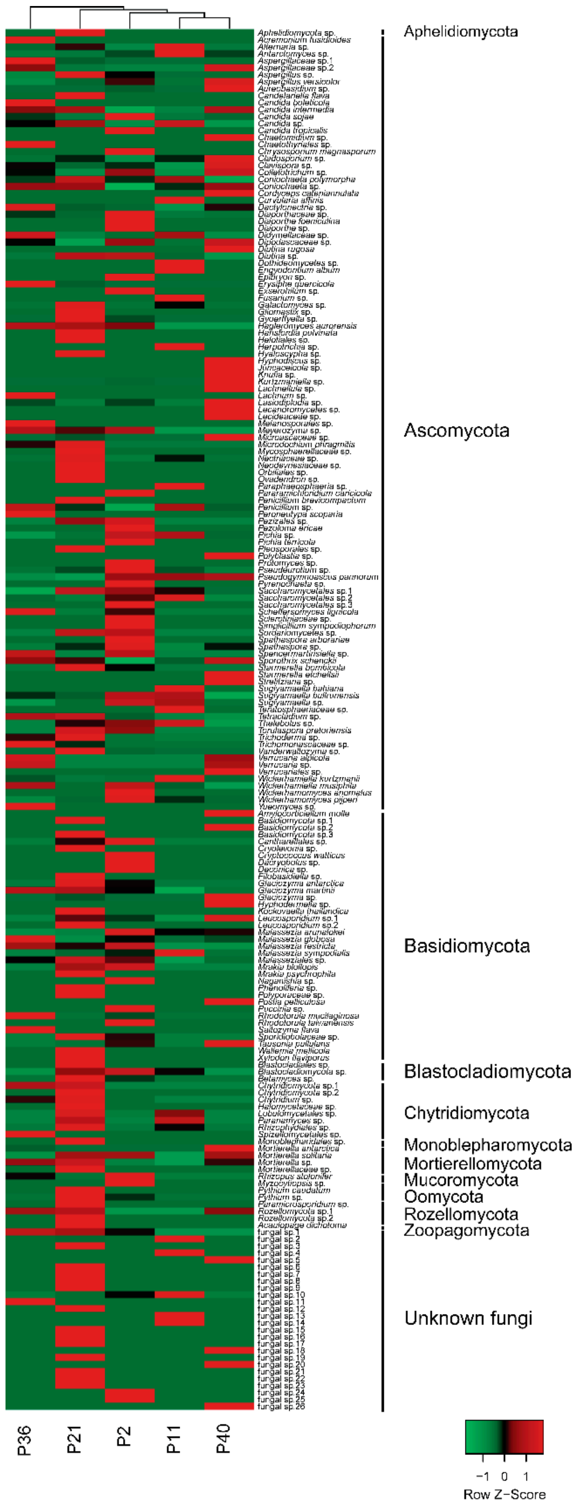

Suppl. Figure S1. Heat map of fungal assemblage relative abundances. The color intensities range from red (highest relative abundance) to green (lowest relative abundance). The values represent percentages of fungal reads obtained from the different rock samples from Lions Rump, King George Island, South Shetland Islands. The heatmap of ASV abundance was created using the following parameters: Average Linkage, Spearman Rank Correlation, and Z-score among samples for each ASV.

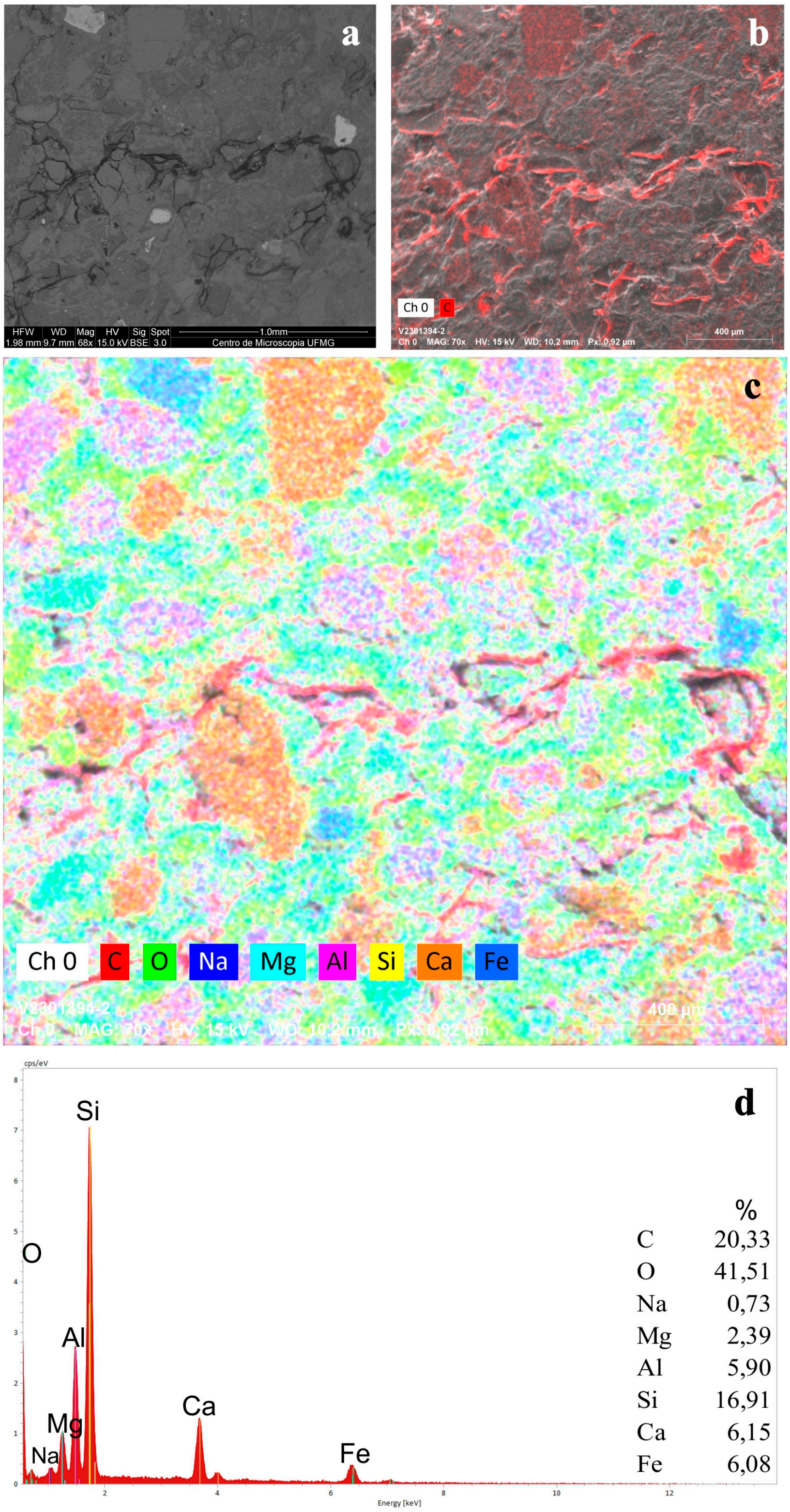

Suppl. Figure S2. Microchemical analyses highlighting (**a**) Backscattered electron image (BSE) obtained under SEM with a predominant void system in sample 36, (**b**) Microchemical map of carbon (red color) marking the voids in the BSE image, (**c**) Microchemical map of the chemical elements C, O, Na, Al, Si, Ca and Fe in different colors and (**d**) Normalized semi-quantitative spectrum of the chemical composition of the microchemical map in image c.

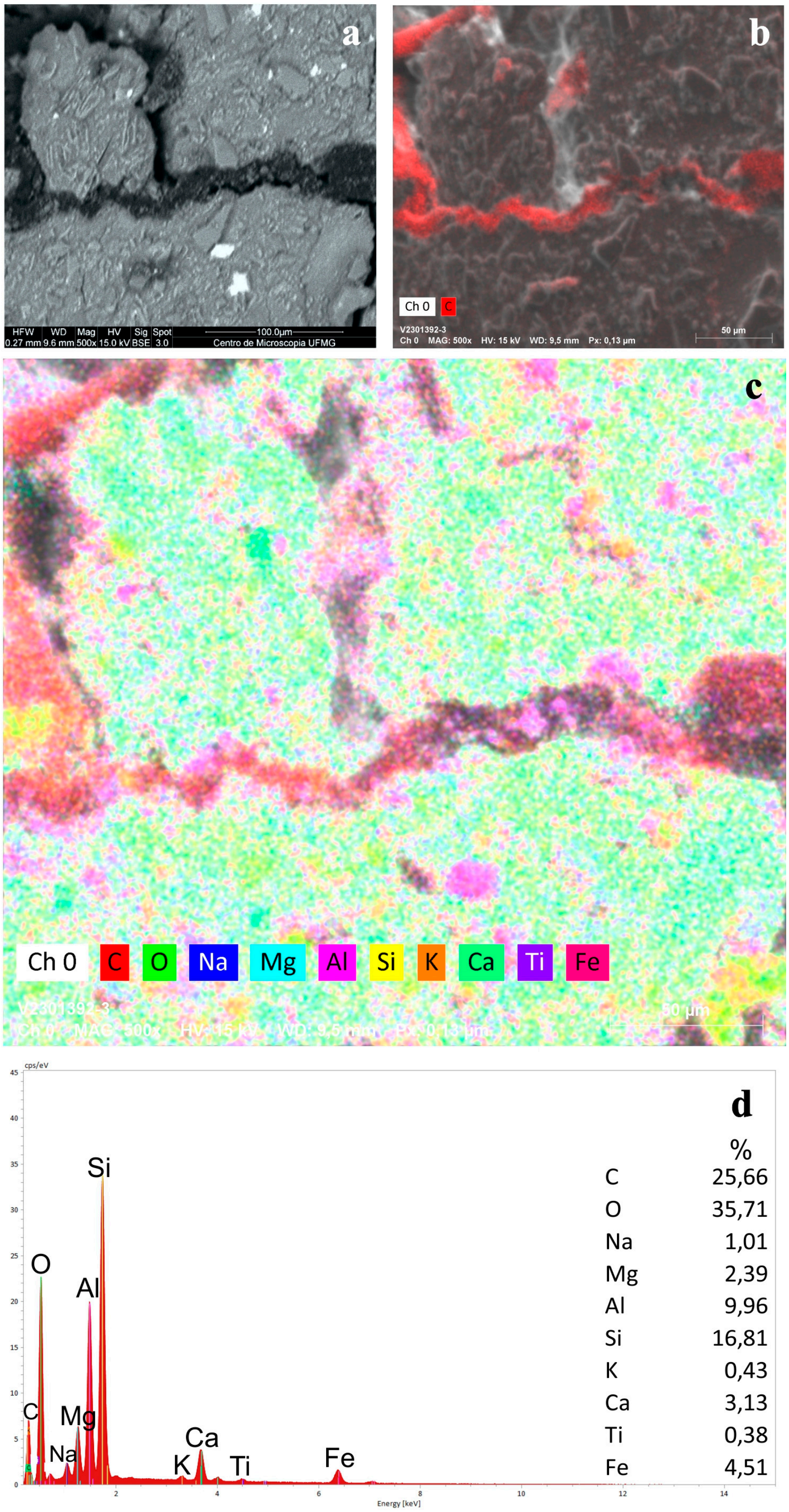

Suppl. Figure S3. Microchemical analyses highlighting (**a**) Backscattered electron image (BSE) obtained under SEM with a predominant void system in sample 40, (**b**) Microchemical map of carbon (red color) marking the voids in the BSE image, (**c**) Microchemical map of the chemical elements C, O,

Na, Al, Si, Ca and Fe in different colors and (**d**) Normalized semi-quantitative spectrum of the chemical composition of the microchemical map in image c.

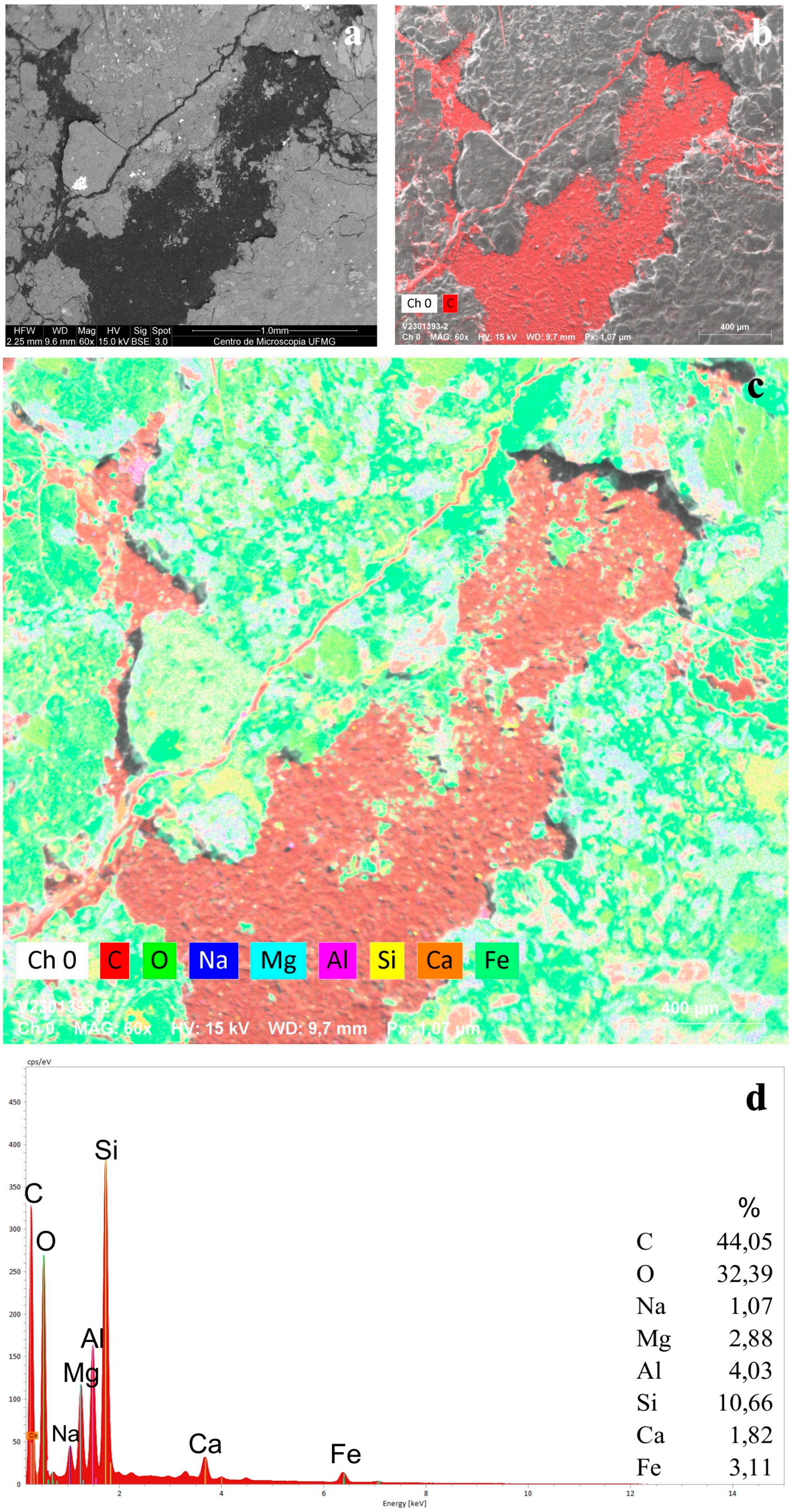

Suppl. Figure S4. Microchemical analyses highlighting (**a**) Backscattered electron image (BSE) obtained under SEM with a predominant void system in sample 11, (**b**) Microchemical map of carbon

(red color) marking the voids in the BSE image, (**c**) Microchemical map of the chemical elements C, O, Na, Al, Si, Ca and Fe in different colors and (**d**) Normalized semi-quantitative spectrum of the chemical composition of the microchemical map in image c.

Suppl. Table S1. Relative abundances of the assigned fungal amplicon sequence variants (ASVs) detected associated with rock samples obtained at Lions Rump, King George Island, South Shetland Islands.

				Relative abundance (%) at the different sites sampled				
**Database**	**Kingdom**	**Phylum**	**Fungal ASV**	**S2**	**S11**	**S21**	**S36**	**S40**
Unite	Fungi	*Ascomycota*	*Pseudogymnoascus pannorum*	28.5137	28.7880	14.7182	17.0139	30.2698
			Penicillium sp.	2.3769	15.4496	9.6695	17.7121	8.7622
			Aspergillus sp.	8.8903	5.2937	20.6203	3.2216	5.5772
			Cladosporium sp.	1.4049	4.4648	4.3810	2.9399	11.3545
			Aspergillaceae sp.	0.4151	0.1496	1.3614	19.1513	0.4498
			Diaporthaceae sp.	13.8820	0.9238	1.9077	3.0789	1.3812
			Saccharomycetales sp.	4.2445	3.7629	4.8186	2.3288	3.2109
			Coniochaeta sp.	1.4631	3.4235	4.5222	4.4188	4.3978
			Dactylonectria sp.	2.5151	0.8453	2.6499	8.0341	3.9625
			Pichia sp.	4.2707	3.9317	2.1293	1.4014	2.2721
			Sugiyamaella sp.	4.7903	4.0718	2.0376	0.9247	1.8853
			Antarctomyces sp.	2.1409	8.0023	1.1955	1.0263	2.3154
			Nectriaceae sp.	1.2894	1.5681	2.5291	1.3540	1.3586
			Pseudeurotium sp.	2.9129	0.6827	0.7538	0.4375	0.3423
			Wickerhamomyces pijperi	1.9485	0.8080	0.6605	0.4343	0.6822
			Wickerhamiella kurtzmanii	0.4191	1.8583	0.3900	0.5669	0.5151
			Tetracladium sp.	0.5062	0.3603	1.0227	0.9210	0.2856
			Coniochaeta polymorpha	0.5227	0.8628	1.0531	0.4840	0.0000
			Strelitziana sp.	0.0000	0.0000	0.0000	0.0000	3.7989
			Curvularia affinis	0.0000	2.7459	0.0000	0.0000	0.0000
			Alternaria sp.	0.0000	1.2886	0.5228	0.2393	0.1312
			Candida sojae	1.1569	0.0000	0.0009	0.1891	0.0786
			Colletotrichum sp.	0.7009	0.2580	0.2723	0.5177	0.9169
			Aspergillus versicolor	0.5631	0.3117	0.3653	0.2042	1.0401
			Sporothrix schenckii	0.1218	0.3715	0.4824	0.5455	0.6799
			Thelebolus sp.	0.3804	0.5946	0.3177	0.1253	0.0133

	Diutina sp.	0.3187	0.2027	0.3115	0.1891	0.1486
	Meyerozyma sp.	0.3978	0.0000	0.2633	0.4029	0.0000
	Erysiphe quercicola	0.0000	0.0000	0.0448	0.8335	0.0000
	Verrucaria alpicola	0.0000	0.0000	0.0000	0.5820	0.3509
	Sugiyamaella bullrunensis	0.2920	0.2761	0.1287	0.1486	0.0000
	Starmerella bombicola	0.1107	0.0000	0.4548	0.0000	0.0000
	Gliomastix sp.	0.0000	0.0000	0.5243	0.0000	0.0000
	Didymellaceae sp.	0.0800	0.2643	0.0313	0.3732	0.0000
	Aureobasidium sp.	0.0582	0.0000	0.0000	0.0000	0.8076
	Verrucaria sp.	0.0000	0.0000	0.0000	0.4275	0.3157
	Pyrenochaeta sp.	0.6467	0.0000	0.0000	0.0000	0.0000
	Candida intermedia	0.0582	0.0813	0.1374	0.1230	0.1405
	Kurtzmaniella sp.	0.0258	0.0000	0.0000	0.0000	0.5434
	Lasiodiplodia sp.	0.0636	0.0000	0.0000	0.0574	0.4122
	Trichoderma sp.	0.0111	0.0000	0.2370	0.0829	0.0000
	Microdochium phragmitis	0.0000	0.0000	0.2426	0.0775	0.0000
	Wickerhamiella musiphila	0.1289	0.0700	0.0664	0.1176	0.0341
	Lachnellula sp.	0.0000	0.0000	0.0000	0.0000	0.5168
	Paraphaeosphaeria sp.	0.0000	0.4636	0.0000	0.0000	0.0000
	Fusarium sp.	0.0000	0.4574	0.0000	0.0000	0.0000
	Gyoerffyella sp.	0.0209	0.0000	0.2229	0.0000	0.0000
	Protomyces sp.	0.3356	0.0000	0.0000	0.0000	0.0000
	Knufia sp.	0.0000	0.0000	0.0000	0.0000	0.4035
	Cordyceps cateniannulata	0.0000	0.0000	0.0000	0.0000	0.3954
	Dipodascaceae sp.	0.0831	0.0491	0.0244	0.0638	0.1006
	Polyblastia sp.	0.0000	0.0000	0.0000	0.0000	0.3723
	Spencermartinsiella sp.	0.1378	0.0000	0.0000	0.1495	0.0000
	Clavispora sp.	0.0507	0.0000	0.0366	0.0624	0.1445
	Verrucariales sp.	0.0000	0.0000	0.0000	0.0000	0.3532
	Aspergillaceae sp.	0.0000	0.0000	0.0103	0.1053	0.1989
	Microascaceae sp.	0.0000	0.0000	0.0257	0.0000	0.2775

	Sordariomycetes sp.	0.0840	0.0000	0.0870	0.0000	0.0000
	Spathaspora sp.	0.1227	0.0000	0.0313	0.0091	0.0347
	Sugiyamaella bahiana	0.0000	0.2524	0.0000	0.0000	0.0000
	Candida sp.	0.0222	0.0621	0.0517	0.0396	0.0197
	Vanderwaltozyma sp.	0.0067	0.0000	0.1302	0.0000	0.0000
	Dothideomycetes sp.	0.0000	0.2355	0.0000	0.0000	0.0000
	Scheffersomyces lignicola	0.0493	0.0000	0.0000	0.1363	0.0000
	Trichomonascaceae sp.	0.0000	0.0000	0.0241	0.1513	0.0000
	Chrysosporium magnasporum	0.1698	0.0000	0.0000	0.0000	0.0000
	Helotiales sp.	0.0000	0.0000	0.1114	0.0000	0.0000
	Acremonium fusidioides	0.0000	0.0000	0.0000	0.1613	0.0000
	Starmerella etchellsii	0.0000	0.0000	0.0000	0.0000	0.1983
	Penicillium brevicompactum	0.0000	0.0000	0.1074	0.0000	0.0000
	Peroneutypa scoparia	0.0000	0.0000	0.0000	0.1549	0.0000
	Pararamichloridium caricicola	0.1471	0.0000	0.0000	0.0000	0.0000
	Hyaloscypha sp.	0.0000	0.0000	0.1005	0.0000	0.0000
	Simplicillium sympodiophorum	0.1427	0.0000	0.0000	0.0000	0.0000
	Orbiliales sp.	0.0000	0.0000	0.0964	0.0000	0.0000
	Epibryon sp.	0.1333	0.0000	0.0000	0.0000	0.0000
	Galactomyces sp.	0.0000	0.0186	0.0786	0.0000	0.0000
	Yueomyces sp.	0.0000	0.0000	0.0000	0.1194	0.0000
	Saccharomycetales sp.	0.0298	0.0632	0.0091	0.0155	0.0058
	Herpotrichia sp.	0.0000	0.1338	0.0000	0.0000	0.0000
	Pleosporales sp.	0.0000	0.0000	0.0733	0.0000	0.0000
	Hansfordia pulvinata	0.0000	0.0000	0.0598	0.0000	0.0000
	Engyodontium album	0.0000	0.1033	0.0000	0.0000	0.0000
	Teratosphaeriaceae sp.	0.0000	0.0994	0.0000	0.0000	0.0000
	Wickerhamomyces anomalus	0.0782	0.0000	0.0000	0.0000	0.0000
	Lachnum sp.	0.0000	0.0000	0.0000	0.0757	0.0000
	Sclerotiniaceae sp.	0.0733	0.0000	0.0000	0.0000	0.0000
	Candida tropicalis	0.0707	0.0000	0.0000	0.0000	0.0000

	Neodevriesiaceae sp.	0.0000	0.0000	0.0407	0.0000	0.0000
	Hyphodiscus sp.	0.0000	0.0000	0.0000	0.0000	0.0728
	Ovadendron sp.	0.0000	0.0000	0.0382	0.0000	0.0000
	Chaetomidium sp.	0.0000	0.0000	0.0000	0.0000	0.0688
	Torulaspora pretoriensis	0.0111	0.0000	0.0203	0.0000	0.0000
	Diutina rugosa	0.0000	0.0000	0.0000	0.0000	0.0335
	Candelariella flava	0.0000	0.0000	0.0163	0.0000	0.0000
	Melanosporales sp.	0.0000	0.0000	0.0000	0.0223	0.0000
	Chaetothyriales sp.	0.0000	0.0000	0.0000	0.0191	0.0000
	Saccharomycetales sp.	0.0187	0.0000	0.0000	0.0000	0.0000
	Candida boleticola	0.0000	0.0000	0.0000	0.0141	0.0000
	Mycosphaerellaceae sp.	0.0000	0.0000	0.0088	0.0000	0.0000
	Diaporthe foeniculina	0.0098	0.0000	0.0000	0.0000	0.0000
	Exserohilum sp.	0.0089	0.0000	0.0000	0.0000	0.0000
	Diaporthe sp.	0.0084	0.0000	0.0000	0.0000	0.0000
	Juncaceicola sp.	0.0000	0.0000	0.0000	0.0000	0.0104
	Pezoloma ericae	0.0076	0.0000	0.0000	0.0000	0.0000
	Spathaspora arborariae	0.0044	0.0000	0.0000	0.0000	0.0000
	Pichia terricola	0.0027	0.0000	0.0000	0.0000	0.0000
Basidiomycota	*Malassezia restricta*	3.1863	1.1841	2.1866	2.7654	1.2147
	Malassezia arunalokei	1.0729	0.3891	0.0520	0.0000	0.4278
	Leucosporidium sp.	0.2947	0.0683	0.4358	0.1372	0.8377
	Malassezia globosa	0.3084	0.1903	0.0648	0.7957	0.2451
	Glaciozyma antarctica	0.2507	0.0000	0.8703	0.0000	0.0000
	Saitozyma flava	0.0000	0.0000	0.0000	1.4196	0.0000
	Malasseziales sp.	0.2191	0.0000	0.3847	0.1796	0.1139
	Malassezia sympodialis	0.1787	0.7397	0.1064	0.0000	0.0607
	Glaciozyma martinii	0.1747	0.0000	0.2830	0.3072	0.1046
	Wallemia mellicola	0.0000	0.0000	0.6173	0.0000	0.0000
	Tausonia pullulans	0.1387	0.0000	0.0119	0.0000	0.4035
	Rhodotorula taiwanensis	0.4147	0.0000	0.0000	0.0000	0.0000

	Amylocorticiellum molle	0.0000	0.0000	0.0000	0.0000	0.4775
	Xylodon flaviporus	0.0000	0.0000	0.2320	0.0000	0.0000
	Polyporaceae sp.	0.0000	0.0000	0.2082	0.0000	0.0000
	Mrakia blollopis	0.1089	0.0000	0.0714	0.0000	0.0000
	Cantharellales sp.	0.1427	0.0000	0.0457	0.0000	0.0000
	Naganishia sp.	0.1947	0.0000	0.0000	0.0000	0.0000
	Glaciozyma sp.	0.0000	0.0000	0.0163	0.0000	0.1833
	Cryptococcus watticus	0.1636	0.0000	0.0000	0.0000	0.0000
	Postia pelliculosa	0.0000	0.0000	0.0000	0.0000	0.1943
	Phenoliferia sp.	0.0000	0.0000	0.1014	0.0000	0.0000
	Kockovaella thailandica	0.0000	0.0000	0.0955	0.0000	0.0000
	Deconica sp.	0.1058	0.0000	0.0000	0.0000	0.0000
	Rhodotorula mucilaginosa	0.0089	0.0000	0.0000	0.0793	0.0000
	Dacryobolus sp.	0.0671	0.0000	0.0000	0.0000	0.0000
	Hyphodermella sp.	0.0000	0.0000	0.0000	0.0000	0.0526
	Filobasidiella sp.	0.0000	0.0000	0.0282	0.0000	0.0000
	Mrakia psychrophila	0.0000	0.0000	0.0241	0.0000	0.0000
	Puccinia sp.	0.0324	0.0000	0.0000	0.0000	0.0000
	Leucosporidium sp.	0.0000	0.0000	0.0207	0.0000	0.0000
	Cryolevonia sp.	0.0000	0.0000	0.0066	0.0000	0.0000
Chytridiomycota	*Lobulomycetales* sp.	0.0631	1.0356	2.1343	0.0000	0.0000
	Rhizophydiales sp.	0.0000	0.5302	1.8814	0.0000	0.0000
	Betamyces sp.	0.2111	0.0000	1.5577	0.0000	0.0000
	Chytridium sp.	0.0591	0.0000	0.5412	0.2010	0.0000
	Paranamyces sp.	0.0000	0.2078	0.1900	0.0000	0.0000
	Halomycetaceae sp.	0.0000	0.0000	0.2454	0.0000	0.0000
	Spizellomycetales sp.	0.0000	0.0000	0.0000	0.1294	0.0000
Mortierellomycota	*Mortierella* sp.	0.1702	0.0000	0.8496	0.6207	0.4810
	Mortierella antarctica	0.0000	0.0000	0.0000	0.1322	2.2553
	Mortierella solitaria	0.4987	0.0000	0.5096	0.0761	0.5128
	Mortierellaceae sp.	0.0000	0.0000	0.2079	0.0000	0.0000

		Rozellomycota	*Rozellomycota* sp.	0.0000	0.0000	0.3522	0.3017	0.2700
			Paramicrosporidium sp.	0.0000	0.0000	0.0692	0.0000	0.0000
		Blastocladiomycota	*Blastocladiomycota* sp.	1.6280	0.7454	1.0900	0.0524	0.0000
			Blastocladiales sp.	0.0000	0.0000	0.0144	0.0000	0.0000
		Monoblepharomycota	*Monoblepharidales* sp.	0.0000	0.0000	0.0085	0.0000	0.0000
		Zoopagomycota	*Acaulopage dichotoma*	0.0000	0.0000	0.0034	0.0000	0.0000
		Mucoromycota	*Rhizopus stolonifer*	0.3173	0.0000	0.0103	0.0916	0.0000
		Aphelidiomycota	*Aphelidiomycota* sp.	0.0000	0.0000	0.0873	0.0000	0.0000
	Stramenopila	*Oomycota*	*Pythium caudatum*	0.0000	0.0000	0.1415	0.0000	0.0000
			Pythium sp.	0.0169	0.0000	0.1052	0.0000	0.0000
			Myzocytiopsis sp.	0.0129	0.0000	0.0000	0.0000	0.0000
Genbank	Fungi	*Ascomycota*	*Hagleromyces aurorensis*	0.1049	0.0000	0.1312	0.1481	0.0000
			Pezizales sp.	0.0800	0.0000	0.0479	0.0000	0.0000
			Lecideaceae sp.	0.0000	0.0000	0.0000	0.0000	0.1532
			Lecanoromycetes sp.	0.0000	0.0000	0.0000	0.0000	0.0653
		Basidiomycota	*Sporidiobolaceae* sp.	0.1702	0.0000	0.5259	0.0000	0.0000
			Basidiomycota sp.	0.0000	0.0000	0.0498	0.0000	0.0000
			Basidiomycota sp.	0.0000	0.0000	0.0000	0.0000	0.0815
			Basidiomycota sp.	0.0000	0.0000	0.0110	0.0000	0.0000
		Chytridiomycota	*Chytridiomycota* sp.	0.0000	0.0000	0.2335	0.1919	0.0000
			Chytridiomycota sp.	0.0000	0.0000	0.0182	0.0000	0.0000
		Rozellomycota	*Rozellomycota* sp.	0.0000	0.0000	0.0269	0.0000	0.0000
		Fungi	Fungal sp.	0.0573	0.0000	0.0955	0.1158	0.0000
		Fungi	Fungal sp.	0.0000	0.3490	0.0056	0.0000	0.0000
		Fungi	Fungal sp.	0.0000	0.0000	0.1953	0.0000	0.0000
		Fungi	Fungal sp.	0.0000	0.1705	0.0000	0.0000	0.0000
		Fungi	Fungal sp.	0.0000	0.0000	0.0000	0.0000	0.1295
		Fungi	Fungal sp.	0.0000	0.0000	0.0701	0.0000	0.0000
		Fungi	Fungal sp.	0.0000	0.0000	0.0645	0.0000	0.0000
		Fungi	Fungal sp.	0.0000	0.0000	0.0563	0.0000	0.0000
		Fungi	Fungal sp.	0.0000	0.0000	0.0210	0.0000	0.0000

Fungi	Fungal sp.	0.0818	0.3230	0.0216	0.0000	0.0000
Fungi	Fungal sp.	0.0000	0.0000	0.0000	0.2693	0.0000
Fungi	Fungal sp.	0.0000	0.0000	0.1249	0.0000	0.0000
Fungi	Fungal sp.	0.0000	0.2225	0.0000	0.0000	0.0000
Fungi	Fungal sp.	0.0000	0.1942	0.0000	0.0000	0.0000
Fungi	Fungal sp.	0.0000	0.0000	0.0513	0.0000	0.0000
Fungi	Fungal sp.	0.0000	0.0000	0.0507	0.0000	0.0000
Fungi	Fungal sp.	0.0000	0.0000	0.0482	0.0000	0.0000
Fungi	Fungal sp.	0.0000	0.0000	0.0000	0.0000	0.0595
Fungi	Fungal sp.	0.0000	0.0000	0.0269	0.0000	0.0000
Fungi	Fungal sp.	0.0000	0.0000	0.0000	0.0000	0.0324
Fungi	Fungal sp.	0.0000	0.0000	0.0175	0.0000	0.0000
Fungi	Fungal sp.	0.0000	0.0000	0.0088	0.0000	0.0000
Fungi	Fungal sp.	0.0000	0.0000	0.0069	0.0000	0.0000
Fungi	Fungal sp.	0.0093	0.0000	0.0000	0.0000	0.0000
Fungi	Fungal sp.	0.0058	0.0000	0.0000	0.0000	0.0000
Fungi	Fungal sp.	0.0000	0.0000	0.0000	0.0000	0.0023

Green indicates dominant, blue intermediate and orange minor relative abundance (see Methods). ASV = amplicon sequence variant.

Suppl. Table S2. fungal lifestyles at generic level obtained from FunGuild[*] and other sources for the taxa detected in the five rock samples analyzed in the current study.

Fungal genera	Relative abundance (%) at the different sites sampled					Trophic mode	FUNGuild
	**S2**	**S11**	**S21**	**S36**	**S40**		
Acaulopage	nr	nr	0.0034	nr	nr	Pathotrophic	Animal Pathogen*
Acremonium	nr	nr	nr	0.1613	nr	Pathotrophic-Saprotrophicic-Symbiotrophic	Animal Pathogen- Endophyte-Fungal Parasite-Plant Pathogen-Wood Saprotrophicic*
Alternaria	nr	1.2886	0.5228	0.2393	0.1312	Pathotrophic-Saprotrophicic-Symbiotrophic	Animal Pathogen-Endophyte-Plant Pathogen-Wood Saprotrophic*
Amylocorticiellum	nr	nr	nr	nr	0.4775	Saprotrophic	Undefined Saprotrophic*
Antarctomyces	2.1409	8.0023	1.1955	1.0263	2.3154	Saprotrophicic	Undefined Saprotrophicic*
Aspergillus	8.8903	5.2937	20.6203	3.2216	5.5772	Pathotrophic-Saprotrophicic	Animal Pathogen-Undefined Saprotrophicic*
Aureobasidium	0.0582	nr	nr	nr	0.8076	Pathotroph-Saprotrophic-Symbiotroph	Animal Pathogen-Endophyte-Epiphyte-Plant Pathogen-Undefined Saprotrophic*
Betamyces	0.2111	nr	1.5577	nr	nr	Saprotrophicic	Freshwater and Soil Saprotrophicic*
Candelariella	nr	nr	0.0163	nr	nr	Symbiotroph	Lichenized*
Candida	0.2616	0.0287	0.0380	0.0732	0.0478	Pathotrophic-Saprotrophicic-Symbiotrophic	Animal Pathogen-Endophyte-Endosymbiont-Epiphyte-Soil Saprotrophic*
Chaetomidium	nr	nr	nr	nr	0.0688	Saprotrophic	Dung Saprotrophic-Undefined Saprotrophic*
Chrysosporium	0.1698	nr	nr	nr	nr	Pathotrophic-Saprotrophicic	Animal Pathogen-Soil Saprotrophic [89]
Chytridium	0.0591	nr	0.5412	0.2010	nr	Pathotroph	Plant Pathogen*
Cladosporium	1.4049	4.4648	4.3810	2.9399	11.3545	Symbiotrophic	Endophyte*

Clavispora	0.0507	nr	0.0366	0.0624	0.1445	Pathotroph	Animal Pathogen*
Colletotrichum	0.7009	0.2580	0.2723	0.5177	0.9169	Pathotroph-Symbiotroph	Endophyte-Plant Pathogen*
Coniochaeta	1.4631	3.4235	4.5222	4.4188	4.3978	Pathotrophic-Saprotrophicic-Symbiotrophic	Animal Pathogen-Dung Saprotrophicic-Endophyte-Lichen Parasite-Plant Pathogen-Undefined Saprotrophic*
Cordyceps	nr	nr	nr	nr	0.3954	Entomopathogen	Arthropod pathogen [90]
Cryolevonia	nr	nr	0.0066	nr	nr	Saprotrophic, mycoparasites and phytoparasites [91]	
Cryptococcus	0.1636	nr	nr	nr	nr	Pathotroph-Saprotrophic-Symbiotroph	Animal Pathogen-Endophyte-Epiphyte-Undefined Saprotrophic*
Curvularia	nr	2.7459	nr	nr	nr	Pathotroph	Plant Pathogen*
Dacryobolus	0.0671	nr	nr	nr	nr	Saprotrophic	Wood Saprotrophic*
Dactylonectria	2.5151	0.8453	2.6499	8.0341	3.9625	Soil Saprotrophicic	Plant Pathogen*
Deconica	0.1058	nr	nr	nr	nr	Saprotrophic	Undefined Saprotrophic*
Diaporthe	0.0098	nr	nr	nr	nr	Pathotroph-Symbiotroph	Endophyte-Plant Pathogen*
Diaporthe	0.0084	nr	nr	nr	nr	Pathotroph-Symbiotroph	Endophyte-Plant Pathogen*
Diutina	0.3187	0.2027	0.3115	0.1891	0.1486	Pathotroph	Humans and animals pathogen [92]
Engyodontium	nr	0.1033	nr	nr	nr	Pathotroph	Animal Pathogen*
Epibryon	0.1333	nr	nr	nr	nr	Pathotroph	Plant Pathogen*
Erysiphe	nr	nr	0.0448	0.8335	nr	Pathotroph	Plant Pathogen [93]
Exserohilum	0.0089	nr	nr	nr	nr	Pathotroph	Plant Pathogen*
Filobasidiella	nr	nr	0.0282	nr	nr	Saprotrophic	Undefined Saprotrophic*
Fusarium	nr	0.4574	nr	nr	nr	Pathotrophic-Saprotrophicic-Symbiotrophic	Animal Pathogen-Endophyte-Lichen, Parasite-Plant Pathogen-Soil, Saprotrophicic-Wood Saprotrophic*
Galactomyces	nr	0.0186	0.0786	nr	nr	Pathotrophic	Plant Pathogen*
Glaciozyma	0.2507	nr	0.8703	0.3072	0.1046	Saprotrophicic	Undefined Saprotrophicic*
Gliomastix	nr	nr	0.5243	nr	nr	Saprotrophic	Undefined Saprotrophic*

Gyoerffyella	0.0209	nr	0.2229	nr	nr	Saprotrophicic	Undefined Saprotrophicic*
Hagleromyces	0.1049	nr	0.1312	0.1481	nr	Pathotroph	Human Pathogen [94]
Hansfordia	nr	nr	0.0598	nr	nr	Saprotrophic	Undefined Saprotrophic*
Herpotrichia	nr	0.1338	nr	nr	nr	Saprotrophic	Undefined Saprotrophic*
Hyaloscypha	nr	nr	0.1005	nr	nr	Saprotrophic	Undefined Saprotrophic*
Hyphodermella	nr	nr	nr	nr	0.0526	Saprotrophic	Undefined Saprotrophic*
Hyphodiscus	nr	nr	nr	nr	0.0728	Pathotroph	Fungal Parasite*
Juncaceicola	nr	nr	nr	nr	0.0104	Symbiotroph	Endophyte [95]
Knufia	nr	nr	nr	nr	0.4035	Pathotroph-Saprotrophic	Animal Pathogen-Plant Pathogen-Soil Saprotrophic-Undefined Saprotrophic*
Kockovaella	nr	nr	0.0955	nr	nr	Symbiotroph	Epiphyte*
Kurtzmaniella	0.0258	nr	nr	nr	0.5434	Symbiotroph [96]	
Lachnellula	nr	nr	nr	nr	0.5168	Saprotrophic	Undefined Saprotrophic*
Lachnum	nr	nr	nr	0.0757	nr	Saprotrophic	Undefined Saprotrophic*
Lasiodiplodia	0.0636	nr	nr	0.0574	0.4122	Pathotroph	Plant Pathogen*
Leucosporidium	0.2947	0.0683	0.4358	0.1372	0.8377	Saprotrophicic	Soil Saprotrophicic-Undefined Saprotrophic*
Malassezia	1.1866	0.6258	0.6024	0.8903	0.4871	Pathotroph-Saprotrophic	Animal Pathogen-Undefined Saprotrophic*
Meyerozyma	0.3978	nr	0.2633	0.4029	nr	Symbiotroph [97]	
Microdochium	nr	nr	0.2426	0.0775	nr	Pathotroph	Plant pathogen - Saprotrophic [98]
Mortierella	0.2230	nr	0.6796	0.2763	1.0830	Saprotrophicic-Symbiotrophic	Endophyte-Litter Saprotrophicic-Soil, Saprotrophicic-Undefined Saprotrophic*
Mrakia	0.1089	nr	0.0714	nr	nr	Saprotrophicic	Soil Saprotrophicic-Undefined Saprotrophic*
Myzocytiopsis	0.0129	nr	nr	nr	nr	Pathotrophic	Animal Pathogen [99]
Naganishia	0.1947	nr	nr	nr	nr	Saprotrophicic	Soil-Flowers*
Ovadendron	nr	nr	0.0382	nr	nr	Pathotrophic	Human pathogen

Paramicrosporidium	nr	nr	0.0692	nr	nr	Parasite [100]	
Paranamyces	nr	0.2078	0.1900	nr	nr	Parasite	Plant parasite [101]
Paraphaeosphaeria	nr	0.4636	nr	nr	nr	Saprotrophic	Undefined Saprotrophic*
Pararamichloridium	0.1471	nr	nr	nr	nr	Saprotrophic [102]	
Penicillium	2.3769	15.4496	9.6695	17.7121	8.7622	Saprotrophicic	Dung Saprotrophicic-Undefined* Saprotrophicic-Wood Saprotrophicic*
Peroneutypa	nr	nr	nr	0.1549	nr	Endophytic or saprobic on plant substrates [103]	
Pezoloma	0.0076	nr	nr	nr	nr	Pathotroph-Saprotrophic-Symbiotroph	Bryophyte Parasite-Ectomycorrhizal-Ericoid Mycorrhizal-Undefined Saprotrophic-Wood Saprotrophic*
Phenoliferia	nr	nr	0.1014	nr	nr	Symbiotroph [104]	
Pichia	4.2707	3.9317	2.1293	1.4014	2.2721	Pathotrophic-Saprotrophicic-Symbiotrophic	Animal-Plant Pathogen- Animal Endosymbiont-Undefined Saprotrophicic*
Polyblastia	nr	nr	nr	nr	0.3723	Symbiotroph	Lichenized*
Postia	nr	nr	nr	nr	0.1943	Saprotrophic [105]	
Protomyces	0.3356	nr	nr	nr	nr	Pathotrophic	Plant Pathogen*
Pseudeurotium	2.9129	0.6827	0.7538	0.4375	0.3423	Saprotrophicic	Undefined Saprotrophicic*
Pseudogymnoascus	28.5137	28.7880	14.7182	17.0139	30.2698	Pathotrophic-Saprotrophicic-Symbiotrophic	Animal pathogen-Soil Saprotrophicic*
Puccinia	0.0324	nr	nr	nr	nr	Pathotrophic	Plant Pathogen [105]
Pyrenochaeta	0.6467	nr	nr	nr	nr	Pathotrophic-Symbiotrophic-Saprotrophicic	Plant Pathogen- Endophyte -Soil* Saprotrophicic*
Pythium	0.0169	nr	0.1415	nr	nr	Pathotroph-Saprotrophic	Pant-Animal Pathogen [106]
Rhizopus	0.3173	nr	0.0103	0.0916	nr	Pathotrophic-Saprotrophicic	Animal Endosymbiont-Animal Pathogen-Endophyte-Plant Pathogen-Undefined Saprotrophic*

Rhodotorula	0.4147	nr	nr	0.0793	nr	Pathotroph-Saprotrophic	Animal Endosymbiont-Animal Pathogen-Endophyte-Plant Pathogen-Undefined Saprotrophic*
Saitozyma	nr	nr	nr	1.4196	nr	Symbiotroph [107]	
Scheffersomyces	0.0493	nr	nr	0.1363	nr	Saprotrophicic-Symbiotrophic [107]	
Simplicillium	0.1427	nr	nr	nr	nr	Pathotroph	Animal Pathogen*
Spathaspora	0.1227	nr	0.0313	0.0091	0.0347	Saprotrophic [109]	
Spencermartinsiella	0.1378	nr	nr	0.1495	nr	Saprotrophicic [110]	
Sporothrix	0.1218	0.3715	0.4824	0.5455	0.6799	Pathotroph-Saprotrophic-Symbiotroph	Animal Pathogen-Endophyte-Plant Saprotrophic-Soil Saprotrophic*
Starmerella	0.1107	nr	0.4548	nr	0.1983	Symbiotrophic	Plant-Animal Symbiotrophic [111]
Strelitziana	nr	nr	nr	nr	3.7989	Pathotroph	Plant Pathogen*
Sugiyamaella	4.7903	4.0718	2.0376	0.9247	1.8853	Saprotrophic-Symbiotroph [112]	
Sugiyamaella	0.2920	0.2524	0.1287	0.1486	nr	Saprotrophic [113]	
Tausonia	0.1387	nr	0.0119	nr	0.4035	Symbiotrophic	Plant Symbiotrophic [114]
Tetracladium	0.5062	0.3603	1.0227	0.9210	0.2856	Saprotrophicic	Undefined Saprotrophicic*
Thelebolus	0.3804	0.5946	0.3177	0.1253	0.0133	Saprotrophicic-Symbiotrophic	Dung Saprotrophicic-Endophyte-Undefined Saprotrophicic*
Torulaspora	0.0111	nr	0.0203	nr	nr	Symbiotrophic [115]	
Trichoderma	0.0111	nr	0.2370	0.0829	nr	Pathotrophic-Saprotrophicic-Symbiotrophic	Animal Pathogen-Endophyte-Epiphyte-Fungal Parasite-Plant Pathogen-Wood Saprotrophicic*
Vanderwaltozyma	0.0067	nr	0.1302	nr	nr	Symbiotrophic [116]	
Verrucaria	nr	nr	nr	0.5820	0.3509	Symbiotrophic	Lichenized [117]
Verrucaria	nr	nr	nr	0.4275	0.3157	Symbiotrophic	Lichenized*
Wallemia	nr	nr	0.6173	nr	nr	Saprotrophic	Undefined Saprotrophic*
Wickerhamiella	0.4191	1.8583	0.3900	0.5669	0.5151	Saprotrophicic [118]	
Wickerhamiella	0.1289	0.0700	0.0664	0.1176	0.0341	Saprotrophicic [118]	

Wickerhamomyces	1.9485	0.8080	0.6605	0.4343	0.6822	Saprotrophicic	Undefined Saprotrophicic*
Xylodon	nr	nr	0.2320	nr	nr	Saprotrophic	Undefined Saprotrophic*

nr = not recorded. [a]Source: [*]FunGuild.
